# Social Determinants of Substance Use in Black Adults with Criminal Justice Contact: Do Sex, Stressors, and Sleep Matter?

**DOI:** 10.3390/ijerph22081176

**Published:** 2025-07-25

**Authors:** Paul Archibald, Dasha Rhodes, Roland Thorpe

**Affiliations:** 1 Department of Social Work, College of Staten Island, City University of New York, Staten Island, NY 10314, USA; 2 School of Social Work, College of Behavioral and Community Sciences, University of South Florida, Sarasota, FL 33620, USA; dasharhodes@usf.edu; 3 Department of Health, Behavior, and Society, Program for Research on Men’s Health, Johns Hopkins Bloomberg School of Public Health, Baltimore, MD 21205, USA; rthorpe@jhu.edu

**Keywords:** stressors, lifetime alcohol abuse, cigarette use, marijuana use, sleep problems, Black adults, criminal justice contact, social determinants of health (SDOH)

## Abstract

Substance use is a critical public health issue in the U.S., with Black communities, particularly those with criminal justice contact, disproportionately affected. Chronic exposure to stressors can lead to substance use as a coping strategy. This study used data from 1476 Black adults with criminal justice involvement from the National Survey of American Life to examine how psychosocial stress and sleep disturbances relate to lifetime substance use and to determine if there are any sex differences. Sex-separate generalized linear models for a Poisson distribution with a log-link function estimated prevalence ratios and adjusted prevalence ratios (APRs) for lifetime alcohol abuse, lifetime cigarette, and marijuana use. Independent variables include stressors (family, person, neighborhood, financial, and work-related) and sleep problems, with covariates such as age, SES, and marital status. Lifetime alcohol abuse was associated with family stressors (APR = 2.72) and sleep problems (APR = 3.36) for males, and financial stressors (APR = 2.75) and sleep problems (APR = 2.24) for females. Cigarette use was linked to family stressors (APR = 1.73) for males and work stressors (APR = 1.78) for females. Marijuana use was associated with family stressors (APR = 2.31) and sleep problems (APR = 2.07) for males, and neighborhood stressors (APR = 1.72) for females. Lifetime alcohol abuse, as well as lifetime cigarette and marijuana use, was uniquely associated with various psychosocial stressors among Black adult males and females with criminal justice contact. These findings highlight the role of structural inequities in shaping substance use and support using a Social Determinants of Health framework to address addiction in this population.

## 1. Introduction

Substance use and addiction remain pressing public health issues in the United States, disproportionately affecting Black communities with a history of criminal justice involvement [1,2,3,4]. A growing body of evidence emphasizes the central role of stress in the transition into substance use [5,6]. Stress not only influences initiation and continued substance use but also serves as a key mechanism through which Social Determinants of Health (SDOH), such as poverty, housing instability, and unemployment, shape behavioral health outcomes [7,8]. Chronic exposure to such stressors can result in neurobiological and behavioral changes that heighten vulnerability to substance use as a maladaptive coping strategy [9].

For individuals with criminal justice contact, particularly Black adults, stress is both a driver and a consequence of social and structural inequities. The cumulative burden of economic strain, residential disadvantages, family disruption, and limited access to healthcare creates a cycle of psychosocial distress that contributes to substance use risk [10,11]. Moreover, stress related to systemic racism, surveillance, police contact, and incarceration compound the effects of poverty and marginalization, elevating the risk of substance use as a means of emotional regulation [12,13].

Along with psychosocial stressors such as financial hardship, family strain, and neighborhood distress, sleep disruption has been consistently linked to substance use, even after adjusting for demographic and socioeconomic characteristics [14,15]. However, Haeny and colleagues have reported sex differences in how Black adults describe their stressors and sleep disturbances, as well as in the substance use triggers they experience [16]. Yet, few studies have explored how these stressors uniquely intersect with substance use behaviors in Black adult males and females who have been involved in the criminal justice system. This population faces distinctive vulnerabilities, including high rates of unemployment, low income, and limited access to supportive resources, which has been shown to influence patterns of alcohol, tobacco, and drug use [1,2,3,4].

Drawing on a Social Determinants of Health framework (SDOH) [17], we investigate how life stressors, including family, financial, personal, neighborhood, work-related stress, and sleep problems, are associated with substance use outcomes. The SDOH framework recognizes that health behaviors and outcomes are not simply the result of individual choices but are shaped by broader social, economic, and environmental contexts [8,13,17]. Researchers have used the SDOH framework to highlight the diverse substance use disorder profiles among individuals, emphasizing the interconnected structural, social, and economic risk factors that shape their experiences with substance use [17]. We hypothesize that among Black adult males and females with criminal justice contact, higher levels of psychosocial stress (e.g., financial, family, personal, neighborhood, and work-related stress), and sleep problems will be positively associated with increased prevalence of lifetime alcohol abuse, cigarette use, and marijuana use. To test this hypothesis, the present study examined the prevalence and correlates of lifetime alcohol abuse, as well as lifetime cigarette and marijuana use, among a national sample of Black adult males and females with a history of criminal justice contact.

## 2. Materials and Methods

### 2.1. Study Population

Data for this study came from the National Survey of American Life (NSAL), which was conducted by the Program for Research on Black Americans (PRBA) at the University of Michigan’s Institute for Social Research. The NSAL aimed to examine racial and ethnic differences in mental disorders, psychological distress, and service utilization, focusing on African American and Caribbean Black populations in the United States compared to White respondents in the same communities. The survey was conducted between February 2001 and March 2003 through face-to-face and some telephone interviews. Participants included a national household probability sample of adults aged 18 and older, with one randomly selected adult from each household. This study achieved an overall response rate of 71%, with African Americans comprising 58% of respondents and non-Hispanic Whites comprising 16% [18]. Despite its age, the NSAL remains the most comprehensive national dataset for studying ethnically diverse Black populations [19]. It is the largest and most extensive survey of its kind, utilizing innovative data collection methods such as the Wide-Area Screening Procedure (WASP), which helped identify Black individuals living in predominantly non-Black areas [20]. The dataset includes extensive measures on work and life stress, with a robust sample of Black respondents. The NSAL employed a multi-stage probability sampling approach, selecting respondents from 252 geographic areas across the United States through a four-step process. Interviews lasted approximately two hours and 20 min and were conducted via laptop-assisted personal interviews in respondents’ homes. To enhance response accuracy, 86% of interviews were conducted by racially/ethnically matched interviewers, while 14% were conducted via telephone [18]. The final sample consisted of 3570 African American, 1438 Black Caribbean, and 891 non-Hispanic White adults. For this study, data were extracted from the public-use NSAL dataset for 3570 Black or African American U.S. citizens, collectively identified as Black adults, who were 18 years or older and had reported experiences of being unfairly stopped, searched, questioned, physically threatened, or abused by police. The dataset also included individuals who had ever been arrested as an adult or juvenile, had ever been in any type of correctional facility, or were currently on parole, with a total subsample of 1,476 meeting these criteria.

### 2.2. Measures

#### 2.2.1. Dependent Variables

Lifetime alcohol abuse was determined using a modified WHO-CIDI, aligned with DSM-IV and ICD criteria. Coded as 1 (meets criteria for lifetime alcohol abuse, diagnosed at any point in a participant’s life, regardless of whether it was currently active) or 0 (does not meet).

Lifetime cigarette use was coded as 1 if participant smoked ≥100 cigarettes in their lifetime; otherwise, 0.

Lifetime marijuana use was coded as 1 if participants reported ever using marijuana and use in the past 12 months; otherwise, 0.

#### 2.2.2. Independent Variables

Each life stressor variable (family, personal, neighborhood, financial, and work-related) and was coded dichotomously, where a value of 1 indicated the presence of the stressor, and 0 indicated its absence. Similarly, the sleep-problems variable was coded dichotomously, where a value 1 indicated the presence of sleep problems, and 0 indicated its absence.

Family stressors were measured by factors assessing familial conflict, perceived support, and family closeness. These items captured the quality and cohesion of the family unit. Participants who endorsed any item indicating family conflict (e.g., frequent arguments, tension, or lack of emotional support) or indicated no support across the perceived support or closeness items were coded as 1 = presence of family stressors. Participants who did not endorse any family conflict and who reported at least some perceived support and closeness were coded as 0 = no family stressors.

Personal stressors included experiences of victimization, legal issues, racial discrimination, and personal or health-related problems, reflecting individualized sources of stress. A participant was coded as 1 if they reported any of these personal stressors; otherwise, they were coded 0 (no reported personal stressors).

Neighborhood stressors were evaluated based on participant reports of inadequate local amenities, discomfort within the neighborhood, perceived crime rates, and the presence of drug activity. Reporting any of these indicators was coded as 1 (presence of neighborhood stressors), and reporting none was coded as 0 (no presence of neighborhood stressors).

Financial stressors encompassed financial hardship, inability to meet financial obligations, worsening financial circumstances, and receipt of food stamps. Reporting any financial difficulty and/or receiving food stamps was coded as 1 (presence of financial stressors); no reported difficulty was coded as 0 (no presence of financial stressors).

Work stressors were measured by assessing job dissatisfaction, job insecurity, perceived workplace discrimination, and working excessive hours. If any of these conditions were reported, participants were coded as 1 (presence of work stressors); otherwise, they were coded as 0 (no presence of work stressors).

Sleep problems were assessed using six indicators: insomnia, oversleeping, sleep-medication use, and reports of restless or poor-quality sleep. Participants reporting any of these were coded as having sleep problems (1); otherwise, they were coded as 0 (no sleep problems).

#### 2.2.3. Covariates

*Demographic variables* included sex (1 = male; 0 = female), age (measured continuously in years), and marital status (1 = married or cohabiting; 0 = not married).

Socioeconomic status (SES) measures included education (coded as 1 for participants with a high school diploma or higher, and 0 for those with less than a high school education), income (coded as 1 for annual income ≥ USD 30,000, and 0 for income < USD 30,000, based on the 2003 median household income for Black Americans at the time of data collection [21], and employment (coded as 1 for employed individuals, and 0 for those unemployed).

### 2.3. Statistical Analysis

Sample characteristics were summarized for the full sample. Rao–Scott chi-square tests and Student’s *t*-tests were conducted to compare Black adult males with criminal justice contact to Black adult females with criminal justice contact. Additionally, the ages of Black adult males with criminal justice contact compared to Black adult females with criminal justice contact were examined using logistic regression in combination with F-tests of statistical significance. Existing medical and public health research has indicated that when an outcome variable has a prevalence of 10% or higher, using prevalence ratio estimations is recommended to reduce the risk of overestimation [22,23]. Therefore, in our initial analyses, generalized linear models (GLMs) for a Poisson distribution with a log-link function and robust variance estimator were used to estimate prevalence ratios for lifetime alcohol abuse and lifetime cigarette and marijuana use, examining associations with family, personal, neighborhood, financial, and work stressors, as well as sleep problems. Additional GLMS for Poisson distribution with a log-link function and robust variance estimator were conducted to estimate the prevalence ratios for lifetime alcohol abuse and lifetime cigarette and marijuana use, incorporating the same stress variables and sleep-problems variable, along with sociodemographic characteristics and socioeconomic status. All analyses accounted for the complex, multistage probability sampling design of the NSAL, using appropriate survey weights and design factors. All statistical analyses were performed using the complex survey design procedures in STATA version 14 [24], with statistical significance set at *p* < 0.05.

## 3. Results

### 3.1. Sample Characteristics

The descriptive characteristics and weighted percentages for the full sample, as well as by sex (male and female) are displayed in Table 1. Among the 1476 Black adult respondents with criminal justice contact, 61.4% reported lifetime marijuana use, 19.1% reported lifetime alcohol abuse, and 56.6% reported lifetime cigarette use. Compared to Black females with criminal justice contact, Black males with criminal justice contact were significantly more likely to report lifetime marijuana use (65.5% vs. 54.6%), lifetime alcohol abuse (21.9% vs. 12.2%), and cigarette use (59.4% vs. 51.9%). Black males with criminal justice contact reported a higher average age (*M* = 43.6; *SD* = 15.6) compared to Black females (*M* = 39.6; *SD* = 14.7).

In contrast, Black females with criminal justice contact were significantly more likely than Black males with criminal justice contact to report an annual income below USD 30,000 (72.5% vs. 45.6%), being unmarried (68.7% vs. 50.7%), and being unemployed (40.2% vs. 30.3%). Black females with criminal justice contact also reported significantly higher levels of sleep problems (51.8% vs. 33.4%), family stressors (48.9% vs. 28.7%), neighborhood stressors (52.8% vs. 45.6%), and financial stressors (72.9% vs. 58.9%) comparative to Black males with criminal justice contact.

### 3.2. Unadjusted Associations

The unadjusted associations between life stressors, sleep status, and lifetime alcohol abuse, as well as lifetime cigarette and marijuana use, among Black adult males with criminal justice contact are shown in Table 2. Black adult males with criminal justice contact who reported lifetime alcohol abuse had a significantly higher prevalence of family stressors (prevalence ratio (PR) = 2.52; 95% confidence interval (CI) [1.73, 3.67]), financial stressors (PR = 1.49; 95% CI [1.06, 2.20]), and sleep problems (PR = 3.12; 95% CI [1.88, 4.13]) compared to those who did not report lifetime alcohol abuse. Black adult males with criminal justice contact who reported lifetime marijuana use also showed significantly higher prevalence of family stressors (PR = 2.38; 95% CI [1.50, 3.78]) and sleep problems (PR = 2.09; 95% CI [1.31, 3.34]) compared to those who did not report lifetime marijuana use. No other significant associations were observed between lifetime alcohol abuse, lifetime cigarette and marijuana use, and the remaining life stressor variables or sleep-problems variable.

The unadjusted associations between life stressors, sleep status, and lifetime alcohol abuse, as well as lifetime cigarette and marijuana use, among Black adult females with criminal justice contact are represented in Table 3. Black adult females with criminal justice contact who reported lifetime alcohol abuse had a significantly higher prevalence of financial stressors (PR = 3,26, 95% CI [1.23, 7.68]) and sleep problems (PR = 2.47, 95% CI [1.49, 4.11]) compared to those who did not report lifetime alcohol abuse. Black adult females with criminal justice contact who reported lifetime marijuana use showed significantly higher prevalence of neighborhood stressors (PR = 1.67, 95% CI [1.15, 2.43]) and sleep problems (PR = 1.61, 95% CI [1.04, 2.49]) compared to those who did not report lifetime marijuana use. No other significant associations were observed between lifetime alcohol abuse, lifetime cigarette and marijuana use, and the remaining life stressor variables or sleep-problems variable.

### 3.3. Adjusted Associations

The adjusted associations among life stressors, sleep status, and lifetime alcohol abuse, as well as lifetime cigarette and marijuana use, among Black adult males with criminal justice contact, controlling for sociodemographic factors and socioeconomic status, are shown in Table 4. Black adults with criminal justice contact who reported lifetime alcohol abuse had a significantly higher prevalence of family stressors (adjusted prevalence ratio (APR) = 2.72, 95% CI [1.89, 3.91]) and sleep problems (APR = 3.36, 95% CI [2.09, 4.40]) compared to those without lifetime alcohol abuse. Additionally, those reporting lifetime alcohol abuse were more likely to be older (APR = 1.03, 95% CI [1.02, 1.05]) compared to their counterparts without lifetime alcohol abuse. Black adult males with criminal justice contact who reported lifetime cigarette use also had a higher prevalence of family stressors (APR = 1.73, 95% CI [1.04, 2.92]) compared to those without lifetime cigarette use. They were also more likely to be older (APR = 1.04, 95% CI [1.02, 1.06]) compared to those without lifetime cigarette use. Among Black adult males with criminal justice contact who reported lifetime marijuana use, there was a significantly higher prevalence of family stressors (APR = 2.31 95% CI [1.40, 3.80]) and sleep problems (APR = 2.07, 95% CI [1.27, 3.40]) compared to those without lifetime marijuana use. Those who reported lifetime marijuana use were also more likely to be younger (APR = 0.97, 95% CI [0.96, 0.99]) compared to those without lifetime marijuana use. No other significant associations were identified between lifetime alcohol abuse, cigarette or marijuana use, and the remaining variables.

The adjusted associations among life stressors, sleep status, and lifetime alcohol abuse, as well as lifetime cigarette and marijuana use, among Black adult females with criminal justice contact, controlling for sociodemographic factors and socioeconomic status, are shown in Table 5. Black adults with criminal justice contact who reported lifetime alcohol abuse had a significantly higher prevalence of financial stressors (adjusted prevalence ratio (APR) = 2.75, 95% CI [1.04, 5.31]) and sleep problems (APR = 2.24, 95% CI [1.36, 3.67]) compared to those without lifetime alcohol abuse. Additionally, those reporting lifetime alcohol abuse were more likely to be older (APR = 1.02, 95% CI [1.01, 1.054]) and less likely to have an income greater than or equal to USD 30,000 (APR = 0.39, 95% CI [0.16, 0.84]) compared to their counterparts without lifetime alcohol abuse. Black adult females with criminal justice contact who reported lifetime cigarette use also had a higher prevalence of work stressors (APR = 1.78, 95% CI [1.10, 2.87]) compared to those without lifetime cigarette use. They were also more likely to be older (APR = 1.05, 95% CI [1.02, 1.07]) and less likely to have an income greater than or equal to USD 30,000 (APR = 0.62, 95% CI [0.40, 0.96]) compared to those without lifetime cigarette use. Among Black adult females with criminal justice contact who reported lifetime marijuana use, there was a significantly higher prevalence of neighborhood stressors (APR = 1.72 95% CI [1.13, 2.61]) compared to those without lifetime marijuana use. Those who reported lifetime marijuana use were also more likely to be younger (APR = 0.98, 95% CI [0.97, 0.99]) compared to those without lifetime marijuana use. No other significant associations were identified between lifetime alcohol abuse, lifetime cigarette or marijuana use, and the remaining variables.

## 4. Discussion

This study examined the associations between life stressors, sleep disturbances, and lifetime alcohol abuse, as well as lifetime cigarette and marijuana use, among Black adults with criminal justice contact. Using a Social Determinants of Health (SDOH) framework, our analysis revealed that lifetime alcohol abuse, as well as lifetime cigarette and marijuana use, was uniquely associated with various psychosocial stressors among Black adult males and females with criminal justice contact. Family stressors emerged as the most consistent predictors of lifetime substance use among Black male adults with criminal justice contact. On the other hand, financial stressors, work stressors, and neighborhood stressors were the predictors of lifetime substance use for Black female adults with criminal justice contact. Although the relationships between stressors and substance use show up differently according to sex, these findings align with previous research showing that social stressors, such as strained family relationships, financial insecurity, work stress, and neighborhood instability, increase vulnerability to substance use as a maladaptive coping strategy [5,15]. For Black adults with criminal justice contact, these stressors reflect cumulative disadvantage, as many face job loss, housing instability, and weakened social support networks after incarceration [10]. Within an SDOH lens, substance use can be viewed as a response to chronic exposure to socioeconomic deprivation and interpersonal instability, conditions that are socially patterned and disproportionately experienced by Black communities. These findings underscore the influence of social and structural conditions, such as economic hardship, family instability, and poor sleep quality, on substance use behaviors among populations disproportionately affected by systemic inequities [25,26].

The strong association between sleep problems and substance use, particularly lifetime alcohol abuse for both black adult males and females with criminal justice and lifetime marijuana use for Black male adults with criminal justice contact, underscores the relevance of applying the SDOH framework. Sleep is not merely an individual health behavior; it is deeply influenced by broader structural conditions, such as environmental stress, neighborhood safety, housing quality, and employment stability, all of which are shaped by racialized social and economic stratification [13,27]. In our sample, financial, neighborhood, and work stressors were significantly associated with substance use among Black females with criminal justice contact, but not among their male counterparts. Additionally, Black females with criminal justice contact who reported annual incomes below USD 30,000 were more likely to report lifetime alcohol abuse and lifetime cigarette use. This points to an intersectional burden rooted in both race- and sex-based disparities [28]. Black women with criminal justice contact face unique and compounded vulnerabilities that often differ from those of their male counterparts [28,29]. These findings are consistent with national data from the time of the study which indicated that Black women earned less than men overall and that single women faced higher poverty rates than married-couple families [30,31]. Importantly, data collection occurred during a period of significant economic strain—the early 2000s’ recession—when unemployment rose steadily and job recovery was slow [32,33]. This economic downturn likely exacerbated financial stress, neighborhood disadvantage, and reduced employment opportunities for Black females, particularly those with a history of criminal justice involvement. Such structural and economic constraints may contribute not only to disrupted sleep but also to increased substance use as a coping mechanism in the face of limited social and material resources.

On the other hand, in this sample of Black males with criminal justice contact, family stressors were significantly associated across all three substance use variables. Also, Black males with criminal justice contact who reported annual incomes below USD 30,000 were more likely to report lifetime cigarette use. These patterns may be explained by the influence of social and economic conditions, such as limited employment opportunities and family disruption resulting from criminal justice involvement, which shape health behaviors and substance use risk [13,34]. For instance, Archibald and Thorpe [2] described how the Environmental Affordances Model suggested that substance use may serve as a coping mechanism for chronic stress in the context of limited access to health-promoting resources [35]. For Black men with criminal justice contact, substance use may reflect adaptive responses to structural constraints, including racial discrimination, social exclusion, and economic deprivation. As previously discussed, these behaviors are not merely individual choices but are shaped by environments that more readily encourage unhealthy coping strategies over healthier alternatives. These findings point to the synergistic nature of stress, sleep disturbance, and substance us, where multiple co-occurring conditions interact and are exacerbated by adverse social conditions [26]. This reinforces the shift away from interpreting results through an individual-level pathology lens and highlights the need to address the socio-structural drivers of substance use among criminal justice-involved populations.

Given the multitude of stressors faced by Black adults in this study, it is likely that substances such as cigarettes, alcohol, and marijuana are used as accessible, though maladaptive, coping mechanisms. Such usage may stem from the cultural mistrust [36] and stigma and inequities associated with seeking medical support, compounded by their criminal justice history and racial identity [37]. The cumulative effects of these discriminatory practices can lead to a heightened vulnerability to substance use [38]. Furthermore, the criminal justice system contributes to racial disparities, with Black individuals disproportionately represented in arrests, convictions, and incarcerations [39]. This overrepresentation exacerbates existing stressors and further limits access to support and resources, thus possibly increasing the risk for substance use.

This study adds to the growing body of literature highlighting the importance of social and structural determinants of substance use among Black populations with criminal justice involvement. The consistent associations between stressors and substance use suggest that addressing upstream factors, such as financial insecurity, family strain, and housing instability, may help prevent or reduce substance use in this high-risk group. Furthermore, sleep health interventions, particularly for Black women in justice-involved contexts, may serve as an effective point of intervention in reducing substance use risk.

A methodological strength of our study is the use of modified Poisson regression to estimate prevalence ratios. This provides a more accurate understanding of high-prevalence outcomes than logistic regression [23]. The use of the NSAL also allows for the examination of culturally relevant stressors within a large, nationally representative sample of Black Americans, a population often underrepresented or homogenized in substance use research.

Despite its strengths, this study is not without limitations. Firstly, the cross-sectional nature of the NSAL data precludes causal inferences, and the reliance on self-report measures may introduce recall or social-desirability biases, and participants may underreport or inaccurately recall their substance use or stress experiences. Additionally, while the NSAL remains one of the most comprehensive datasets for studying Black Americans, the data were collected over two decades ago. Given the substantial social, economic, and policy shifts that have occurred since the data were gathered, some findings may not fully capture current dynamics. However, the study offers important historical context regarding the persistent challenges faced by Black Americans with criminal justice contact. Also, the structural inequities and psychosocial burdens faced by Black individuals with criminal justice contact remain salient today [10,11], suggesting that our findings continue to hold relevance.

The generalizability of the findings is also limited by the study’s focus on a specific subsample, Black adults with criminal justice involvement, which may not reflect the broader experiences of Black populations or those from other racial and ethnic groups. However, to address these limitations, future research should consider longitudinal designs to better capture the relationships between stress, sleep, and substance use over time. Expanding study samples to include more diverse populations may also enhance generalizability and help explore how these associations vary across demographic, social contexts, and time. Moreover, integrating qualitative approaches can shed light on the lived experiences underlying these statistical associations, including culturally specific stressors, coping mechanisms, and social supports.

## 5. Conclusions

In conclusion, our findings underscore the utility of the SDOH framework for understanding substance use among Black adults with criminal justice contact, with a consideration of sex differences. Stress, sleep, and substance use are not isolated phenomena but seem to be rooted in structural and social inequities. A deeper understanding of the role of intersectionality, as well as the intersection between stress exposure and substance use, is critical for informing policies and practices that aim to reduce structural inequities and improve behavioral health outcomes within this population. Expanding access to culturally informed and responsive support services specific to those with criminal justice contact may help reduce reliance on substances as a coping mechanism and contribute to improved overall health and well-being. From a policy and prevention standpoint, the SDOH framework calls for interventions that go upstream. Addressing only the symptoms (e.g., substance use) without targeting the root causes (e.g., poverty and neighborhood segregation) limits the effectiveness of traditional substance use prevention strategies. Our findings suggest that improving economic stability and strengthening social supports may be more impactful than stand-alone behavioral interventions. We must also consider including an intersectional examination of stressors and sleep problems, specifically race and sex, in our substance use assessments.

## Figures and Tables

**Table 1:** Descriptive characteristics of Black adults with criminal justice contact for the total sample and by sex, 2001–2003 National Survey of American Life.

	Total Sample N	Male ^a^ N (wt%)	Female ^a^ N (wt%)	*p*-Value ^b^
Total	1476	788 (62.1%)	688 (37.9%)	
Lifetime Alcohol Abuse				
Yes	260 (19.1)	180 (21.9)	80 (12.2)	**<** **0** **.001**
Lifetime Cigarette Use				
Yes	811 (56.6)	472 (59.4)	339 (51.9)	**0.008**
Lifetime Marijuana Use				
Yes	859 (61.4)	503 (65.5)	356 (54.6)	**0.013**
Education				
≥HS	422 (29.9)	243 (31.2)	179 (27.8)	0.269
<HS	1054 (70.1)	545 (68.8)	509 (72.2)	
Income				
≥USD 30,000	552 (44.2)	389 (54.4)	163 (27.5)	**<** **0** **.001**
<USD 30,000	924 (55.8)	399 (45.6)	525 (72.5)	
Marital Status				
Married	520 (42.5)	340 (49.3)	180 (31.3)	**<** **0** **.001**
Work Status				
Employed	947 (66.0)	539 (69.7)	408 (59.8)	**0.009**
Sleep Status				
Sleep Problems	609 (40.4)	261 (33.4)	348 (51.8)	**<** **0** **.001**
Family Stressors				
Stressed	558 (36.4)	225 (28.7)	333 (48.9)	**<** **0** **.001**
Personal Stressors				
Stressed	247 (17.0)	123 (16.0)	124 (18.7)	0.310
Neighborhood Stressors				
Stressed	733 (48.4)	366 (45.6)	367 (52.8)	**0.027**
Financial Stressors				
Stressed	957 (64.2)	462 (58.9)	495 (72.9)	**<** **0** **.001**
Work-Related Stressors				
Stressed	804 (57.1)	440 (57.9)	364 (55.9)	0.519
Age (Mean and SD)	41.8 (15.3)	43.6 (15.6)	39.6 (14.7)	**0.007**

^a^ Column percentages by sex, not percentage of the whole sample. ^b^ Rao–Scott Chi-square *p*-value indicates whether the distribution of sex is significantly different by sub-group. Abbreviations: N, sample size; wt%, weighted percentages; HS, high school; SD, standard deviation. Bold numbers indicate a statistically significant correlation.

**Table 2:** Unadjusted associations between life stressors and sleep status as predictors of the likelihood of lifetime alcohol abuse, and lifetime cigarette and marijuana use among Black adult males with criminal justice contact, 2001–2003 National Survey of American Life (N = 788).

Variable	Lifetime Alcohol Abuse		Lifetime Cigarette Use		Lifetime Marijuana Use	
	PR	95% CI	PR	95% CI	PR	95% CI
Life Stressors						
Family Stressor	**2.52**	**1.73**, **3.67**	1.57	0.95, 2.57	**2.38**	**1.50**, **3.78**
Personal Stressor	0.62	0.32, 1.19	1.04	0.61, 1.76	1.67	0.86, 3.22
Neighborhood Stressor	1.15	0.69, 1.92	1.14	0.74, 1.75	0.75	0.45, 1.24
Financial Stressor	**1.49**	**1.06**, **2.20**	1.34	0.91, 1.98	1.26	0.80, 1.97
Work Stressor	1.02	0.58, 1.71	1.17	0.78, 1.78	1.41	0.97, 2.05
Sleep Status						
Sleep Problems	**3.12**	**1.88**, **4.13**	1.08	0.73, 1.60	**2.09**	**1.31**, **3.34**

Abbreviations: N, sample size; PR, prevalence ratio; CI, confidence interval. Bold numbers indicate a statistically significant correlation.

**Table 3:** Unadjusted associations between life stressors and sleep status as predictors of the likelihood of lifetime alcohol abuse, and lifetime cigarette and marijuana use among Black adult females with criminal justice contact, 2001–2003 National Survey of American Life (N = 688).

Variable	Lifetime Alcohol Abuse		Lifetime Cigarette Use		Lifetime Marijuana Use	
	PR	95% CI	PR	95% CI	PR	95% CI
Life Stressors						
Family Stressor	0.90	0.52, 1.41	1.15	0.69, 1.92	1.19	0.79, 1.79
Personal Stressor	1.51	0.86, 2.64	1.31	0.84, 2.03	1.50	0.88, 2.57
Neighborhood Stressor	1.47	0.84, 2.58	1.01	0.73, 1.41	**1.67**	**1.15**, **2.43**
Financial Stressor	**3.26**	**1.23**, **7.68**	1.38	0.82, 2.33	0.96	0.55, 1.67
Work Stressor	0.89	0.49, 1.62	1.23	0.81, 1.77	**1.61**	**1.04**, **2.49**
Sleep Status						
Sleep Problems	**2.47**	**1.49**, **4.11**	1.43	0.96, 2.13	1.24	0.78, 1.96

Abbreviations: N, sample size; PR, prevalence ratio; CI, confidence interval. Bold numbers indicate a statistically significant correlation.

**Table 4:** Adjusted associations between life stressors and sleep status as predictors of the likelihood of lifetime alcohol abuse, and lifetime cigarette and marijuana use among Black adult males with criminal justice contact, 2001–2003 National Survey of American Life (N = 788).

Variable	Lifetime Alcohol Abuse		Lifetime Cigarette Use		Lifetime Marijuana Use	
	APR	95% CI	APR	95% CI	APR	95% CI
Life Stressors						
Family Stressor	**2.72**	**1.89**, **3.91**	**1.73**	**1.04**, **2.92**	**2.31**	**1.40**, **3.80**
Personal Stressor	0.63	0.32, 1.25	1.24	0.72, 2.14	1.46	0.72, 2.93
Neighborhood Stressor	1.11	0.69, 1.79	1.13	0.74, 1.74	0.74	0.44, 1.23
Financial Stressor	1.47	0.96, 2.26	1.21	0.80, 1.83	1.44	0.88, 2.34
Work Stressor	1.26	0.71, 2.24	1.46	0.96, 2.22	1.23	0.85, 1.79
Sleep Status						
Sleep Problems	**3.36**	**2.09**, **4.40**	1.14	0.80, 1.62	**2.07**	**1.27**, **3.40**
Sociodemographics						
Age	**1.03**	**1.02**, **1.05**	**1.04**	**1.02**, **1.06**	**0.97**	**0.96**, **0.99**
Married	0.68	0.41, 1.12	0.99	0.60 1.62	0.90	0.60, 1.34
Socioeconomic Status						
Education						
≥HS Graduate	1.04	0.70, 1.54	0.85	0.55, 1.30	1.27	0.77, 2.09
Income						
≥USD 30,000	0.65	0.38, 1.11	**0.52**	**0.32**, **0.83**	1.27	0.88, 1.82
Employed	1.55	0.96, 2.51	1.32	0.77, 2.27	1.34	0.85, 2.12

Abbreviations: N, sample size; APR, adjusted prevalence ratio; CI, confidence interval; HS, high school. Bold numbers indicate a statistically significant correlation.

**Table 5:** Adjusted associations between life stressors and sleep status as predictors of the likelihood of lifetime alcohol abuse, and lifetime cigarette and marijuana use among Black adult females with criminal justice contact, 2001–2003 National Survey of American Life (N = 688).

Variable	Lifetime Alcohol Abuse		Lifetime Cigarette Use		Lifetime Marijuana Use	
	APR	95% CI	APR	95% CI	APR	95% CI
Life Stressors						
Family Stressor	0.98	0.58, 1.64	1.31	0.78, 2.20	1.12	0.74, 1.69
Personal Stressor	1.35	0.79, 2.30	1.25	0.80, 1.96	1.54	0.89, 2.67
Neighborhood Stressor	1.41	0.83, 2.39	1.03	0.71, 1.51	**1.72**	**1.13, 2.61**
Financial Stressor	**2.75**	**1.04**, **5.31**	1.18	0.68, 2.05	1.04	0.58, 1.84
Work Stressor	1.13	0.61, 2.09	**1.78**	**1.10**, **2.87**	1.35	0.82, 2.23
Sleep Status						
Sleep Problems	**2.24**	**1.36**, **3.67**	1.34	0.87, 2.07	1.32	0.94, 2.08
Sociodemographics						
Age	**1.02**	**1.01**, **1.04**	**1.05**	**1.02**, **1.07**	**0.98**	**0.97**, **0.99**
Married	1.08	0.59, 1.99	1.43	0.90, 2.27	1.04	0.66, 1.65
Socioeconomic Status						
Education						
≥HS Graduate	1.37	0.74, 2.54	0.89	0.60, 1.32	1.34	0.92, 1.90
Income						
≥USD 30,000	**0.39**	**0.16**, **0.84**	**0.62**	**0.40**, **0.96**	1.14	0.64, 2.02
Employed	0.96	0.42, 2.21	1.06	0.70, 1.61	0.94	0.59, 1.50

Abbreviations: N, sample size; APR, adjusted prevalence ratio; CI, confidence interval; HS, high school. Bold numbers indicate a statistically significant correlation.

## Data Availability

Data from the National Survey of American Life (NSAL) were made available through the Inter-University Consortium for Political and Social Research (ICPSR) at the University of Michigan (http://www.icpsr.umich.edu/).

## Abbreviations

The following abbreviations are used in this manuscript:

SDOH	Social Determinants of Health
NSAL	National Survey of American Life
PRBA	Program for Research on Black Americans
WASP	Wide-Area Screening Procedure
WHO-CIDI	World Health Organization Composite International Diagnostic Interview
DSM-IV	*Diagnostic and Statistical Manual of Mental Disorders, Fourth Edition*
SES	Socioeconomic status
WRS	Work-related stress
M	Mean
SD	Standard deviation
N	Sample size
Wt%	weighted percentages
HS	High school
CI	Confidence interval
PR	Prevalence ratio
APR	Adjusted prevalence ration
